# Correction: Application of co-culture technology of epithelial type cells and mesenchymal type cells using nanopatterned structures

**DOI:** 10.1371/journal.pone.0244008

**Published:** 2020-12-09

**Authors:** The PLOS ONE Staff

In the Funding statement, the grant number from the funder National Research Foundation (MSIT) is listed incorrectly. The correct grant number is: 2020R1A2B5B03002154. The complete, correct Funding statement is as follows: This research was supported by the Bio & Medical Technology Development Program of the National Research Foundation funded by the MSIP (NRF-2016M3A9B4919616, NRF-2019M3A9H1103331) and by a grant (20000325) from the Technology Innovation Program funded from the Ministry of Trade, Industry and Energy (MOTIE), and National Research Foundation grant (MSIT) (No. 2020R1A2B5B03002154) funded by the Republic of Korea government. T&R Biofab Co. Ltd and Amorepacific Corporation did not provide financial support, and did not have any roles in this study. The authors belonging to T&R Biofab and Amorepacific companies recently moved to the companies, and no funds have been contributed by the companies.

The publisher apologizes for the error.
