# Supplementary figures and images for: Application of co-culture technology of epithelial type cells and mesenchymal type cells using nanopatterned structures
Source: PLoS One. 2020 May 11;15(5):e0232899. doi: 10.1371/journal.pone.0232899 (PMC7213697; doi:10.1371/journal.pone.0232899)

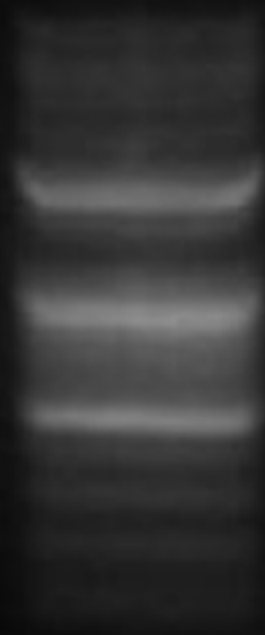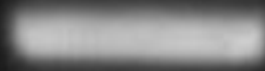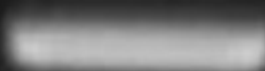

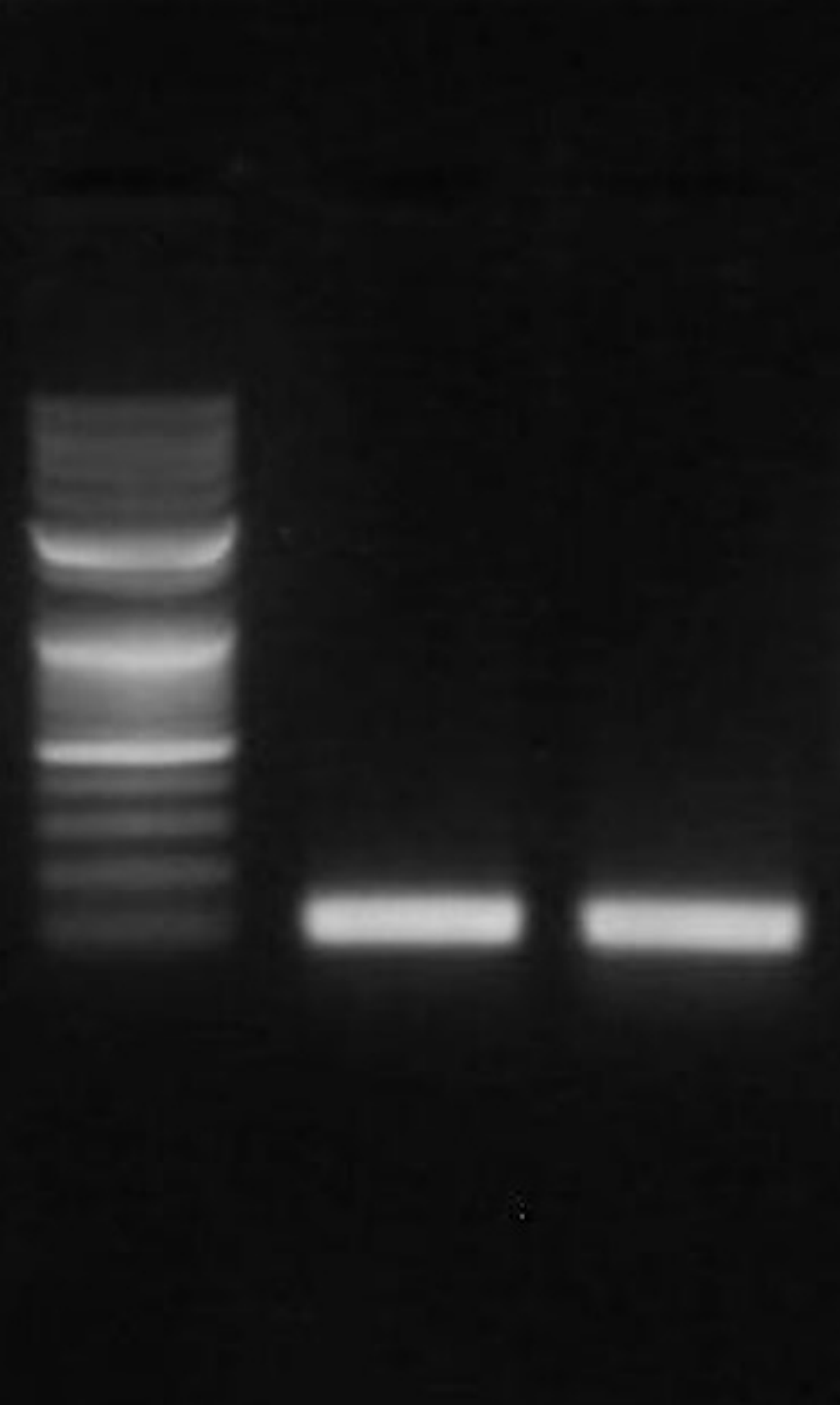



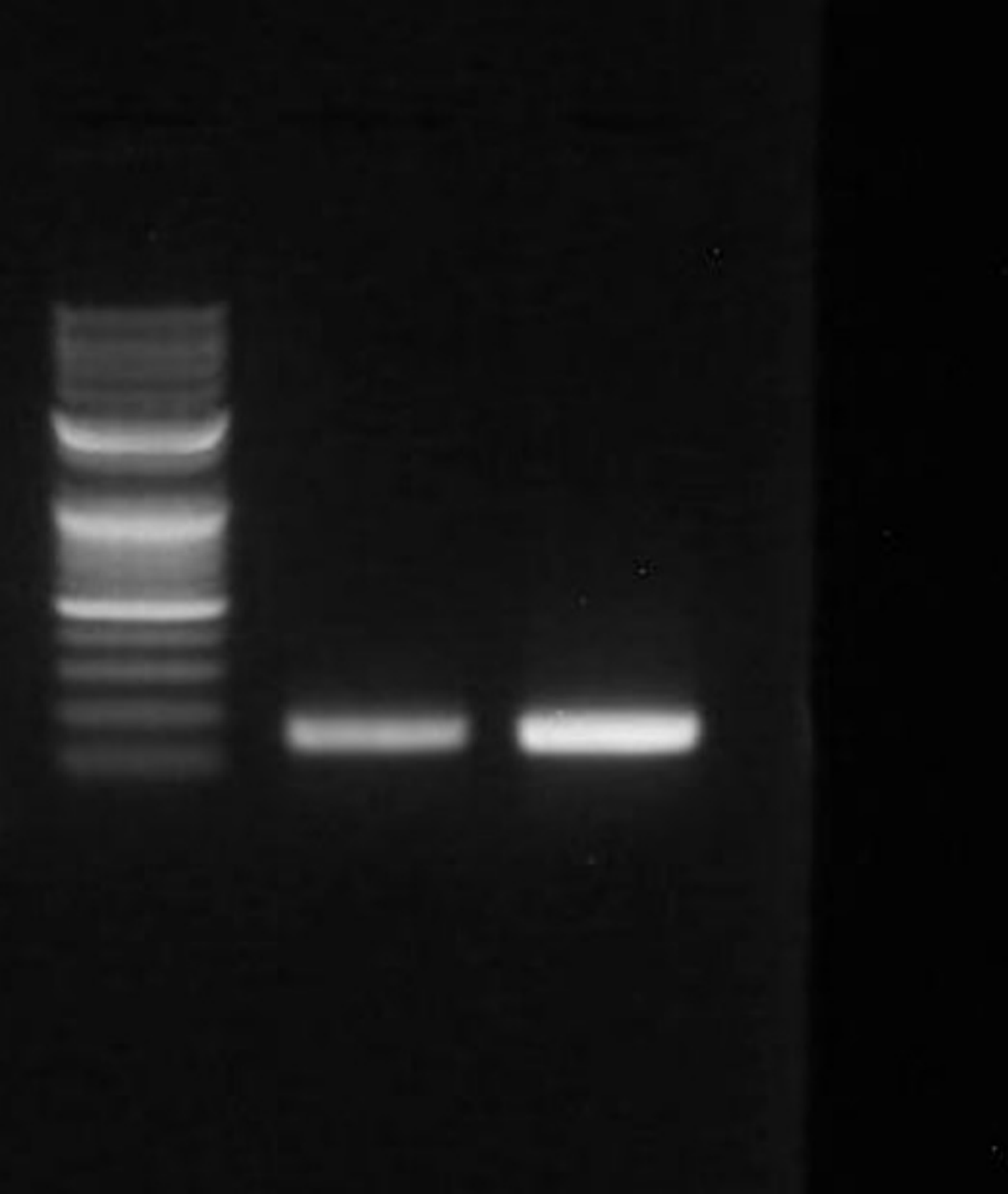

Supplement: S1 Raw images — (PDF) [file pone.0232899.s012.pdf]
