# Supplementary material for: Application of co-culture technology of epithelial type cells and mesenchymal type cells using nanopatterned structures
Source: PLoS One. 2020 May 11;15(5):e0232899. doi: 10.1371/journal.pone.0232899 (PMC7213697; doi:10.1371/journal.pone.0232899)
Supplement: Attachment — Submitted filename: Response to Reviewers.docx [file pone.0232899.s013.docx]

**Response to Journal & Reviewer**

**Manuscript ID:** PONE-D-19-35930

**Manuscript Title:** Application of co-culture technology of epithelial type cells and mesenchymal type cells using nanopatterned structures

**The authors thank the editor and the reviewer for thoughtful comments.**

- **Journal Requirements:**

**1. Thank you for including your competing interests statement;** "The authors have declared that no competing interests exist." We note that one or more of the authors are employed by a commercial company: "T&R Biofab Co. Ltd"; and "Amorepacific Corporation"

**1.1 Funding**

Please provide an amended Funding Statement declaring this commercial affiliation, as well as a statement regarding the Role of Funders in your study.

**1.2 Competing Interests**

Please also provide an updated Competing Interests Statement declaring this commercial affiliation along with any other relevant declarations relating to employment, consultancy, patents, products in development, or marketed products, etc.

**Answer:** T&R Biofab Co. Ltd and Amorepacific Corporation did not provide financial support, and did not have any roles in this study. The authors belonging to T&R Biofab and Amorepacific companies recently moved to the companies, and no funds have been contributed by the companies.

1.1 Funding

This research was supported by the Bio & Medical Technology Development Program of the National Research Foundation funded by the MSIP (NRF-2016M3A9B4919616, NRF-2019M3A9H1103331) and by a grant (20000325) from the Technology Innovation Program funded from the Ministry of Trade, Industry and Energy (MOTIE), and National Research Foundation grant (MSIT) (No. 2017R1A2A1A05001090) funded by the Republic of Korea government.

1.2 Competing Interests

The authors have declared that no competing interests exist. T&R Biofab Co. Ltd and Amorepacific Corporation do not alter our adherence to PLOS ONE policies on sharing data and materials.

**2. PLOS requires an ORCID iD for the corresponding author in Editorial Manager.**

**Answer:** The corresponding author updated the ORCID iD in online system.

**3. PLOS ONE now requires that authors provide the original uncropped and unadjusted images underlying all blot or gel results reported in a submission’s figures or Supporting Information files.**

**Answer:** Original images of Figure 2E were uploaded through ‘Fig 2E_raw_images’.

- **Reviewer Comments:**

Reviewer #1: This study firstly used the nanopattern technology to co-culture mesenchymal cells and epithelial cells. They found that the nanopattern substrates could inhibit the proliferative rate of fibroblasts, but do not influence the growth of keratocytes. Importantly, the application of this technology did not affect the metabolism ability of fibroblasts. They also performed molecule mechanisms underlying these observations. They found that more fibroblasts were arrested in G1 phase and the gene expression of CDKNB1 was upregulated in nanopatterned dishes, comparing to the flat dishes.

Overall, the article is completed and justified. If the authors can carefully revise the English writing throughout the paper and submit high-quality pictures, this manuscript can be considered to be accepted.

**Answer:** We updated the high quality images, and English writing was revised by a native speaker.
